# Supplementary material for: Multifaceted regulation of hepatic lipid metabolism by YY1
Source: Life Sci Alliance. 2021 Jun 7;4(7):e202000928. doi: 10.26508/lsa.202000928 (PMC8200296; doi:10.26508/lsa.202000928)
Supplement: Supplementary file 1 [file LSA-2020-00928_TableS1.docx]

**Supplementary Tables:**

**Table S1:** Summary statistics of RNA-seq data in this study

| sample | condition | batch | reads | reads after trimming | fragments | paired  fragments | paired  fragments% | multi-paired  fragments | multi-paired  fragments% | feature  Counts | feature  Counts% |
| --- | --- | --- | --- | --- | --- | --- | --- | --- | --- | --- | --- |
| R201 | control | batch1 | 6,043,474 | 5,743,724 | 2,871,862 | 2,525,623 | 87.94% | 288,225 | 10.04% | 1,995,095 | 78.99% |
| R202 | control | batch1 | 7,512,238 | 7,083,890 | 3,541,945 | 3,096,125 | 87.41% | 378,359 | 10.68% | 2,471,264 | 79.82% |
| R203 | control | batch1 | 8,472,854 | 8,016,210 | 4,008,105 | 3,456,293 | 86.23% | 474,955 | 11.85% | 2,718,310 | 78.65% |
| R204 | control | batch1 | 8,114,990 | 7,584,578 | 3,792,289 | 3,233,281 | 85.26% | 484,263 | 12.77% | 2,520,344 | 77.95% |
| R209 | control | batch2 | 7,167,654 | 6,693,200 | 3,346,600 | 2,921,920 | 87.31% | 356,999 | 10.67% | 2,308,672 | 79.01% |
| R210 | control | batch2 | 7,057,498 | 6,649,234 | 3,324,617 | 2,826,975 | 85.03% | 429,509 | 12.92% | 2,231,967 | 78.95% |
| R211 | control | batch2 | 6,937,914 | 6,489,772 | 3,244,886 | 2,764,293 | 85.19% | 410,627 | 12.65% | 2,177,959 | 78.79% |
| R216 | YY1-KD | batch1 | 7,329,014 | 7,049,590 | 3,524,795 | 3,144,480 | 89.21% | 335,006 | 9.50% | 2,490,173 | 79.19% |
| R217 | YY1-KD | batch1 | 6,174,486 | 5,804,820 | 2,902,410 | 2,601,010 | 89.62% | 254,647 | 8.77% | 1,986,054 | 76.36% |
| R218 | YY1-KD | batch1 | 8,519,710 | 8,109,416 | 4,054,708 | 3,645,605 | 89.91% | 348,588 | 8.60% | 2,781,909 | 76.31% |
| R219 | YY1-KD | batch2 | 7,623,420 | 7,288,434 | 3,644,217 | 3,213,052 | 88.17% | 370,119 | 10.16% | 2,448,003 | 76.19% |
| R220 | YY1-KD | batch2 | 7,612,524 | 7,193,512 | 3,596,756 | 3,139,303 | 87.28% | 402,623 | 11.19% | 2,442,891 | 77.82% |
| R221 | YY1-KD | batch2 | 8,699,746 | 8,414,754 | 4,207,377 | 3,718,897 | 88.39% | 424,016 | 10.08% | 2,859,700 | 76.90% |
| R222 | YY1-KD | batch3 | 8,201,910 | 7,797,938 | 3,898,969 | 3,536,684 | 90.71% | 306,028 | 7.85% | 2,737,449 | 77.40% |
| R223 | YY1-KD | batch3 | 7,948,610 | 7,400,372 | 3,700,186 | 3,355,631 | 90.69% | 289,207 | 7.82% | 2,610,830 | 77.80% |
| R224 | control | batch3 | 6,442,818 | 6,019,822 | 3,009,911 | 2,693,728 | 89.50% | 254,308 | 8.45% | 2,047,385 | 76.01% |
| R225 | control | batch3 | 6,535,288 | 6,166,572 | 3,083,286 | 2,762,076 | 89.58% | 261,717 | 8.49% | 2,145,306 | 77.67% |
| R226 | control | batch3 | 6,203,910 | 5,737,460 | 2,868,730 | 2,558,782 | 89.20% | 256,491 | 8.94% | 2,009,852 | 78.55% |
| R230 | control | batch4 | 7,293,746 | 6,865,192 | 3,432,596 | 3,078,116 | 89.67% | 290,164 | 8.45% | 2,401,570 | 78.02% |
| R231 | control | batch4 | 6,535,096 | 6,180,356 | 3,090,178 | 2,789,506 | 90.27% | 242,120 | 7.84% | 2,174,946 | 77.97% |
| R232 | control | batch4 | 6,849,206 | 6,643,954 | 3,321,977 | 2,943,123 | 88.60% | 312,885 | 9.42% | 2,271,731 | 77.19% |
| R233 | control | batch4 | 5,492,174 | 4,992,080 | 2,496,040 | 2,262,443 | 90.64% | 185,610 | 7.44% | 1,775,327 | 78.47% |

reads: The number of reads that each sample have in RNA-seq.

reads after trimming: The number of reads left after trimming.

fragments: The number of fragments composed of paired reads after trimming.

paired fragments: The number of fragments that are aligned concordantly and exactly 1 time by HISAT2.

paired fragments%: The percentage of properly paired fragments.

multi-paired fragments: The number of fragments that aligned concordantly > 1 times by HISAT2.

multi-paired fragments%: The percentage of multi-paired fragments.

featureCounts: The number of properly aligned fragments that are mapped to known genes.

featureCounts%: The percentage of the properly aligned fragments that are mapped to known genes.
