# Supplementary material for: Multifaceted regulation of hepatic lipid metabolism by YY1
Source: Life Sci Alliance. 2021 Jun 7;4(7):e202000928. doi: 10.26508/lsa.202000928 (PMC8200296; doi:10.26508/lsa.202000928)
Supplement: Supplementary file 3 [file LSA-2020-00928_TableS3.docx]

**Supplementary Tables:**

**Table S3**: The detailed RT-qPCR validation results on selected genes

| gene | RNA-seq | RT-qPCR  (RSP18) | RT-qPCR  (*P* value) | group |
| --- | --- | --- | --- | --- |
| ACAA2 | -0.70 | -0.80 | 8.7E-12 | DOWN |
| ACADSB | -0.82 | -0.73 | 5.3E-4 | DOWN |
| ACAT1 | -1.03 | -0.84 | 1.73E-12 | DOWN |
| ACAT2 | -1.47 | -1.22 | 4.16E-6 | DOWN |
| ACOX2 | -1.35 | -1.70 | 4.30E-11 | DOWN |
| ACSL4 | -1.10 | -1.23 | 1.07E-07 | DOWN |
| ANGPTL4 | -1.75 | -3.23 | 2.36E-06 | DOWN |
| APOA1 | -1.12 | -1.46 | 3.40E-04 | DOWN |
| APOA5 | -2.01 | -2.02 | 7.86E-07 | DOWN |
| APOB | -1.12 | -0.84 | 7.09E-13 | DOWN |
| APOC3 | -1.46 | -2.10 | 9.18E-07 | DOWN |
| CHREBP | -1.24 | -1.20 | 4.25E-7 | DOWN |
| DHCR7 | -1.07 | -0.75 | 4.31E-7 | DOWN |
| ECH1 | -1.10 | -1.19 | 1.19E-13 | DOWN |
| EHHADH | -1.26 | -1.29 | 3.80E-12 | DOWN |
| ELOVL2 | -3.29 | -2.20 | 2.77E-09 | DOWN |
| ELOVL6 | -1.23 | -1.16 | 6.91E-07 | DOWN |
| FABP1 | -1.99 | -1.81 | 1.73E-07 | DOWN |
| FABP5 | -1.64 | -1.58 | 4.10E-11 | DOWN |
| FADS1 | -1.10 | -1.01 | 2.96E-08 | DOWN |
| FADS2 | -2.40 | -2.10 | 4.22E-06 | DOWN |
| FASN | -1.03 | -0.64 | 0.03 | DOWN |
| FDFT1 | -1.17 | -1.02 | 5.32E-6 | DOWN |
| FOXA1 | -1.10 | -0.79 | 2.94E-05 | DOWN |
| FXR | -2.15 | -1.85 | 9.20E-14 | DOWN |
| HMGCR | -1.41 | -1.20 | 5.99E-6 | DOWN |
| HMGCS1 | -1.84 | -1.71 | 5.11E-6 | DOWN |
| MCEE | -0.72 | -0.56 | 1.04E-5 | DOWN |
| NCOA1 | -1.23 | -1.18 | 1.62E-16 | DOWN |
| NCOA2 | -0.81 | -0.59 | 6.7E-4 | DOWN |
| NCOA3 | -1.76 | -1.73 | 3.12E-17 | DOWN |
| PEX11A | -0.94 | -0.92 | 2.08E-7 | DOWN |
| PGC1A | -0.75 | -1.00 | 1.39E-09 | DOWN |
| PGC1B | -4.33 | -3.46 | 1.12E-14 | DOWN |
| PLTP | -1.01 | -1.22 | 8.28E-07 | DOWN |
| PPARA | -0.70 | -0.91 | 6.12E-08 | DOWN |
| SCD | -1.30 | -1.21 | 4.33E-09 | DOWN |
| SLC27A2 | -0.93 | -1.21 | 9.10E-5 | DOWN |
| SQLE | -1.63 | -1.25 | 3.21E-10 | DOWN |
| SREBF2 | -0.98 | -0.69 | 5.05E-7 | DOWN |
| YY1 | -2.43 | -3.03 | 3.55E-17 | DOWN |
| ACTB | -0.08 | 0.01 | 0.86 | NC |
| ELOVL5 | 0.57 | 0.06 | 0.57 | NC |
| FOXA2 | -0.16 | -0.51 | 0.0035 | NC |
| GAPDH | -0.39 | -0.49 | 4.67E-10 | NC |
| HNF4A | -0.47 | -1.03 | 5.93E-04 | NC |
| LXRA | 0.33 | -0.14 | 0.048 | NC |
| MLX | -0.02 | -0.20 | 0.07 | NC |
| PPARD | 0.53 | 0.46 | 1.34E-05 | NC |
| PPARG | 0.39 | 0.07 | 0.32 | NC |
| RXRA | -0.56 | -0.66 | 1.81E-06 | NC |
| RXRB | 0.21 | -0.12 | 0.44 | NC |
| SREBF1 | -0.58 | -0.37 | 0.07 | NC |
| VDR | 1.86 | -0.19 | 0.39 | UP |
| AKT1S1 | 1.12 | 0.79 | 2.46E-7 | UP |
| CDKN1A | 1.67 | 0.55 | 0.02 | UP |
| CDKN2A | 2.94 | 1.69 | 1.09E-08 | UP |
| CPT1A | 1.18 | -0.65 | 1.50E-05 | UP |
| DEPTOR | 1.98 | 1.61 | 1.86E-5 | UP |
| LXRB | 0.72 | -0.21 | 0.32 | UP |
| TGFB2 | 3.47 | 3.03 | 3.45E-09 | UP |

The log2 fold changes determined in RNA-seq and RT-qPCR (normalized to RSP18) are shown here for each gene. The *P* values for RT-qPCR are shown here which are calculated by two-tailed Student’s t tests. The group shows whether the target genes were upregulated (UP), downregulated (DOWN) or unchanged in RNA-seq.
