# Supplementary material for: Multifaceted regulation of hepatic lipid metabolism by YY1
Source: Life Sci Alliance. 2021 Jun 7;4(7):e202000928. doi: 10.26508/lsa.202000928 (PMC8200296; doi:10.26508/lsa.202000928)
Supplement: Supplementary file 4 [file LSA-2020-00928_TableS4.docx]

**Supplementary Tables:**

**Table S4:** Primers used for gene quantification in RT-qPCR

| **Genes** | **Accession no.** | **Forward primer sequence (5’-3’)** | **Reverse primer sequence (5’-3’)** | **Product size (bp)** |
| --- | --- | --- | --- | --- |
| RSP18 | NM_022551.3 | CGAGTACTCAACACCAACATCG | TGTCTGCTTTCCTCAACACCAC | 109 |
| GAPDH | NM_001289745.3 | GAAGGTGAAGGTCGGAGTCAAC | CAGAGTTAAAAGCAGCCCTGGT | 71 |
| ACTB | NM_001101.5 | TGCCGACAGGATGCAGAAGG | CCGATCCACACGGAGTACTTGC | 99 |
| ELOVL2 | NM_017770.4 | CTTGGAATCACACTTCTCTCCG | GCTACCCGGATGTCAGCTTC | 122 |
| YY1 | NM_003403.5 | GGTGCAGATCAAGACCCTGGA | TCAACCACTGTCTCATGGTCA | 90 |
| FADS1 | NM_013402.7 | ACCCAGACATCAACATGCATC | GCTGGTGGTTGTACGGCATA | 102 |
| FADS2 | NM_004265.4 | GCATGGCATTGAATACCAGGAG | AGGTAGGCGTCCAGCCACA | 99 |
| FXR | NM_001206977.2 | GACTCAAGAGGAGTATGCTCTG | ACATCAAGAAGTGGCTCCTG | 111 |
| LXRA | NM_005693.4 | GGACCAGCTCCAGGTAGAGA | CGGAGGCTCACCAGTTTCAT | 126 |
| LXRB | NM_007121.6 | CCTGAAGGCATCCACTATCG | GTGGAAGTCGTCCTTGCTGT | 115 |
| PPARG | NM_001354666.1 | GGAATTAGATGACAGCGACT | AGCAGGTTGTCTTGAATGTC | 111 |
| PPARA | NM_005036.5 | GTGGCTGCTATCATTTGCTG | CTCTGCAGGTGGAGTCTGAG | 107 |
| PPARD | NM_001171818.1 | GTGACCTGGCCCTATTCATTG | GTTGGCCTGCAGGTGGAATT | 128 |
| CPT1A | NM_001876.4 | AGACCCCTCAGCAGCAAGTG | ACCATAGCCGTCATCAGCA | 101 |
| PLTP | NM_001242920.1 | GGTGCAGATCCCACTACCTG | CCCTTTGGCAAAGTGGAGAT | 103 |
| APOA1 | NM_000039.2 | CAGCGGCAGAGACTATGTGT | CACGCTGTCCCAGTTGTCAA | 88 |
| APOA5 | NM_052968.4 | GGAGCAGATCCATCAGCAGA | GAGGTCTTGCTCAAGGCTGT | 70 |
| APOC3 | NM_000040.3 | CATGAAGCACGCCACCAAGA | GGAACTGAAGCCATCGGTCA | 103 |
| ACSL4 | NM_004458.2 | TCGAGAAGCTGCAAATGCCA | ATGGTTCCTCAGCTCCTTCC | 139 |
| FABP5 | NM_001444.3 | ATTGGTTCAGCATCAGGAGT | ATCCGAGTACAGGTGACATT | 111 |
| ANGPTL4 | NM_139314.3 | ACGATGGCTCAGTGGACTTC | CGTGATGCTATGCACCTTCTC | 107 |
| SCD | NM_005063.5 | ACCTCTTCGGATATCGTCCT | GGAGTGGTGGTAGTTGTGGA | 104 |
| ELOVL5 | NM_021814.5 | GCCGTGCACATTCCCTCTTG | GTGGTCCTTCAGGTGGTCTT | 139 |
| ELOVL6 | NM_024090.2 | ACAATGGACCTGTCAGCAAA | GTGGTGATACCAGTGCAGGA | 125 |
| FASN | NM_004104.5 | GCGGACTACAACCTCTCCCA | GCTGTGGATGATGCTGATGA | 120 |
| PGC1A | NM_013261.5 | CGCAGAGAGTATGAGAAGCGA | GTCCCTCAGTTCTGTCCGTG | 135 |
| PGC1B | NM_133263.4 | GGCGCTTTGAAGTGTTTGGTG | GCGTGCTCAGAACACCGGTAG | 103 |
| ACOX2 | NM_003500.4 | GGCAAGAACAGCCTACCTGGA | GCGTTCGTAGACGTTTCCATC | 133 |
| RXRA | NM_002957.6 | GCGCTGAGGGAGAAGGTCTAT | CAGGCATTTGAGCCCGATGGA | 129 |
| CDKN2A | NM_058195.4 | GGGGCACCAGAGGCAGTAA | CCCGAGGTTTCTCAGAGCC | 99 |
| FABP1 | NM_001443.2 | TGGGGGAGGAATGTGAGCTG | TCGCCGTTGAGTTCGGTCA | 127 |
| CDKN1A | NM_000389.4 | GGAGACTCTCAGGGTCGAAA | GCGGATTAGGGCTTCCTCTT | 98 |
| TGFB2 | NM_003238.4 | GGATGCGGCCTATTGCTTTA | GAAGTTGGCATTGTACCCTT | 124 |
| HNF4A | NM_178849.3 | GGGACCGGATCAGCACTCGA | GCCCGAATGTCGCCGTTGAT | 133 |
| FOXA1 | NM_004496.5 | GCATGAAACCAGCGACTGGAA | CCAGGCCTGAGTTCATGTTG | 89 |
| FOXA2 | NM_021784.5 | GTATGCTGGGAGCGGTGAAG | CATGTTGCTCACGGAGGAGT | 95 |
| SREBF1 | NM_004176.4 | CCGCTCCTCCATCAATGACA | GCAGAAAGCGAATGTAGTCGA | 116 |
| VDR | NM_000376.3 | CAGTTACAGCATCCAAAAGGT | GGCACTTGACTTCAGCAGTAC | 100 |
| DEPTOR | NM_022783.4 | GGAAGTAAGCCATGCCACAT | TGAGCCCGTTGACAGAGAC | 100 |
| AKT1S1 | NM_032375.5 | TGAGCCCACAGAGACAGAG | GGCGTCCTCATCCATCACA | 82 |
| RXRB | NM_001270401.2 | CCAACCCTAGTGAGGTGGAG | GTAGCAGCAGCTTGGCAAAC | 111 |
| CHREBP | NM_032951.3 | CCAGCGTTTTGACCAGATGC | GGCCGGATGAGGATGCTGAA | 99 |
| MLX | NM_198205.2 | CTGGATCGAGGAGCACTGTA | CCGGTCAGTAAAGCTGGTTT | 89 |
| SREBF2 | NM_004599.4 | AGCTGCCAAGGAGAGTCTA | CCTGAGGTTTCACCAAGGAC | 122 |
| NCOA1 | NM_003743.5 | GCTCTCATCCACTGACCTTC | CTGTACACTGGACGTCAGCA | 89 |
| NCOA2 | NM_001321703.2 | GGAGGCAACCTGTTCCCAA | CTGGCTTCAGCAGTGTCAG | 96 |
| NCOA3 | NM_181659.3 | GGCAGCAGTGGTCACATGG | GAGGTCCTGGTGCAGAGAT | 107 |
| ACAT1 | NM_000019.4 | ACGGGCTAACTGATGTCTAC | GCGTCCTGTTCATTTCGTGC | 94 |
| ACAT2 | NM_005891.3 | GCTGTCTCTGCTGCAATAGT | TCGACAGCCAGATGCTCCAA | 108 |
| HMGCS1 | NM_001098272.3 | GGAGTAGGACTTGTGCATTC | TGTGGCAGGGAGTCTTGGTA | 81 |
| HMGCR | NM_000859.3 | CATTGGCAGCAGGACATCTT | AAGCTCCTTGGAGGTCTTGT | 81 |
| FDFT1 | NM_001287742.2 | CACCAATATGCCAGCTGTC | GCTAGAAGATGGGTCTGAGT | 88 |
| SQLE | NM_003129.4 | CAGATGATTCCCTGCATCAAC | GCAACAGCAAAGAAGTGTCC | 142 |
| DHCR7 | NM_001360.3 | ATCCTTGGCTATGCCGTCTC | CGATGCCCATCATGTAGTTG | 115 |
| APOB | NM_000384.3 | GGGCATGGATATGGATGAAGA | CCGGACCCTCAACTCAGTTT | 112 |
| ACAA2 | NM_006111.3 | GGGAGGATCTGGATCAAGAA | CAAGCTGATCCAACGGCATA | 84 |
| ACADSB | NM_001609.4 | CCCTGTGGAGAAATACTTCCG | CGATATGCTTTGCAATGGTG | 92 |
| ECH1 | NM_001398.3 | ACCAAGGTCAACCTGCTGT | ACCGACTTCACGAGGTCTT | 110 |
| MCEE | NM_032601.4 | GTCAACCTGGGAAATACCAAG | TTCAAATCCATCACAGCTGCA | 149 |
| EHHADH | NM_001966.4 | CTTCGCTGAAAGGAAAGCAA | CTCGGCCCATTGTTCCCAA | 116 |
| PEX11A | NM_003847.3 | CCTTTGGGCTAGAAGCCATG | CATGCATGTGTACTGAGTGG | 95 |
| SLC27A2 | NM_003645.4 | CAGCACATTGCTGATTACCTAC | AGGGTCATTTTGCGGTGTT | 104 |
