# Supplementary material for: Multifaceted regulation of hepatic lipid metabolism by YY1
Source: Life Sci Alliance. 2021 Jun 7;4(7):e202000928. doi: 10.26508/lsa.202000928 (PMC8200296; doi:10.26508/lsa.202000928)
Supplement: Supplementary file 6 [file LSA-2020-00928_TableS6.docx]

**Supplementary Tables:**

**Table S6:** Antibodies used in Western blot in this study

| **Antibodies** | **Source** | **Identifier** |
| --- | --- | --- |
| Mouse monoclonal anti-β-actin | Santa Cruz Biotechnology | Cat# sc-81178, RRID:AB_2223230 |
| Rabbit polyclonal anti-YY1 | Santa Cruz Biotechnology | Cat# sc-1703, RRID:AB_2218501 |
| Rabbit monoclonal anti-FADS1 | Abcam | Cat# ab126706, RRID:AB_11130088 |
| Rabbit polyclonal anti-FADS2 | Santa Cruz Biotechnology | Cat# sc-98480, RRID:AB_2278074 |
| Rabbit monoclonal anti-ELOVL2 | Abcam | Cat# ab176327 |
| Rabbit polyclonal anti-SCD | Thermo Fisher Scientific | Cat# PA5-19682, RRID:AB_10982251 |
| Rabbit polyclonal anti-CHREBP | Novus | Cat# NB400-135, RRID:AB_10002435 |
| Rabbit polyclonal anti-SREBF1 | Santa Cruz Biotechnology | Cat# sc-8984, RRID:AB_2194223 |
| Rabbit polyclonal anti-SREBF2 | Santa Cruz Biotechnology | Cat# sc-5603, RRID:AB_2194247 |
| Rabbit polyclonal anti-FXR | Santa Cruz Biotechnology | Cat# sc-13063, RRID:AB_2155051 |
| Rabbit polyclonal anti-PGC1A | Santa Cruz Biotechnology | Cat# sc-13067, RRID:AB_2166218 |
| Mouse monoclonal anti-FOXA1 | Santa Cruz Biotechnology | Cat# sc-514695 |
| Mouse monoclonal anti-FOXA2 | Santa Cruz Biotechnology | Cat# sc-374376, RRID:AB_10989742 |
| Mouse monoclonal anti-HNF4A | Santa Cruz Biotechnology | Cat# sc-374229, RRID:AB_10989766 |
| Rabbit polyclonal anti-RXRA | Santa Cruz Biotechnology | Cat# sc-553, RRID:AB_2184874 |
| Goat polyclonal anti-PPARA | Santa Cruz Biotechnology | Cat# sc-1985, RRID:AB_2165740 |
| Mouse monoclonal anti-PPARA | Santa Cruz Biotechnology | Cat# sc-398394, RRID:AB_2885073 |
