# Supplementary material for: Multifaceted regulation of hepatic lipid metabolism by YY1
Source: Life Sci Alliance. 2021 Jun 7;4(7):e202000928. doi: 10.26508/lsa.202000928 (PMC8200296; doi:10.26508/lsa.202000928)
Supplement: Supplementary file 7 [file LSA-2020-00928_TableS7.docx]

**Supplementary Tables:**

**Table S7:** Primers used in ChIP-qPCR in this study

| **Target** | **Name** | **Forward primer sequence (5’-3’)** | **Reverse primer sequence (5’-3’)** | **Product size (bp)** |
| --- | --- | --- | --- | --- |
| PPARA promoter | P_rep1 | GCTCTCCTACTCCTCGCCATT | GCTGACTTTGTGCCTACGCAA | 89 |
| PPARA promoter | P_rep2 | GGCTAATCAACAAGGGTGAGT | GGACCCACTCCGGAAAGCAAA | 110 |
| PPARA enhancer 1 | E1 | TGGCGGATGACACCCTGGAA | GCCTGCGAAGTTTGCTGCT | 90 |
| PPARA enhancer 2 | E2 | AGAGGAGCAGGGACTCACTA | GCCTAGCAACAGAAGGTGGT | 94 |
| PPARA enhancer 3 | E3 | GTCCGTGGGGAGCTAACTGG | TTCCCCATGGAGGGCATCTT | 107 |
| PPARA enhancer 4 | E4 | CTGACAGCCAGGGAGCAGCA | GGCACTGACCATGGCGTGAC | 152 |
| FABP1 promoter |  | GGAATCAACAGCTGCTGACCT | CCGACCAGACTGTCCACTGTA | 85 |
| SCD promoter |  | CGAGCCAATGGCAACGGCAG | TGGGGAAATGCTAATGAGGCT | 93 |
| ELOVL6 promoter |  | CGCCCTGCAAGCTGAAACTTC | GCGCAGAGAGAAAGGAAGTGG | 98 |
| Negative control | GDCHR12 | GGGATCAGCGCTACTAACTCCT | GACGTAGTTACTATTACGCCCAT | 112 |
